# Commentary: reducing the world's stillbirths

**DOI:** 10.1186/1471-2393-9-S1-S1

**Published:** 2009-05-07

**Authors:** Robert L Goldenberg, Elizabeth M McClure, José M Belizán

**Affiliations:** 1 Department of Obstetrics and Gynecology, Drexel University College of Medicine, Philadelphia, PA 19102, USA; 2 Department of Epidemiology, University of North Carolina at Chapel Hill, Chapel Hill, NC, USA; 3 Institute for Clinical Effectiveness and Health Policy, Buenos Aires, Argentina

## Abstract

One of the major success stories of modern obstetrics in high-income countries in the last 5 decades is the reduction of stillbirths from rates as high as 50 per 1000 births to about 5 per 1000 births today. Fetal mortality associated with obstructed labour, asphyxia, hypertension, diabetes, Rh disease, placental abruption, post-term pregnancies and infections such as syphilis all have declined. Much of this success has occurred in term births in the intrapartum period so that most stillbirths in high-income countries now occur in the antepartum period and are pre-term. Current stillbirth rates in many low- and middle-income countries, and especially in those areas within the countries with poorly functioning health systems, approximate those seen in high-income countries 50 years ago. A major difference between the stillbirths occurring in high-income countries and those occurring elsewhere is the preponderance of late pre-term, term and intrapartum stillbirths in low-resource countries. Those stillbirths should be relatively easy to prevent by known risk assessment methods and prompt delivery, often by Cesarean section. This commentary addresses an extensive six-paper review of stillbirths with an emphasis on low- and middle-income countries. Among the conclusions are that while a number of interventions have been shown to be effective in reducing stillbirths, unless there is a functioning health system in which these interventions can be implemented, the potential for a sustainable and substantial reduction in stillbirth rates will not be reached.

## Commentary

One of the major success stories of modern obstetrics in high-income countries is the reduction of stillbirths. Rates as high as 50 per 1000 births or more were common 40–50 years ago, but are now often less than 5 per 1000 births – nearly a ten-fold reduction [1]. Exactly why this reduction has occurred is not completely clear, but it is almost certainly related to the nearly universal availability of antenatal and intrapartum care that focuses on risk-identification and reduction, and treatment of obstetric complications as they arise. Fetal mortality associated with obstructed labour, asphyxia, hypertension, diabetes, Rh disease, placental abruption, post-term pregnancies and infections such as syphilis has declined. Many of the interventions that treat these conditions have never been studied individually regarding their impact on stillbirth rates, but their collective introduction over the last 50 years appears to have resulted in the impressive reduction in stillbirths described above.

However, reductions in stillbirth rates have not been uniform across all gestational ages, or types of stillbirth. In high-income countries, it is now very uncommon for stillbirths to occur at term, or in the intrapartum period, so that most stillbirths now occur antenatally and are pre-term. In fact, 50% or more of the stillbirths occur prior to 28 weeks gestation [2]. Despite the historical successes, in recent years, the downward trajectory of stillbirth rates in high-income countries has nearly ceased. Consequently, a number of research efforts are underway to understand the recent lack of progress and to develop new interventions that will contribute to further reductions in stillbirth. To achieve success in high-income countries, these interventions will need to reduce stillbirths that occur during the antenatal period and in pre-term fetuses.

Current stillbirth rates in many low- and middle-income countries, and especially those areas within the countries with poorly functioning health systems, approximate those seen in high-income countries 50 years ago [3-6]. A major difference between the stillbirths occurring in high-income countries and those occurring elsewhere is the preponderance of late pre-term, term and intrapartum stillbirths [7-10]. Those stillbirths should be relatively easy to prevent by known risk assessment methods and prompt delivery, often by Cesarean section [7,8,11,12]. Providing the components of modern obstetric care as practiced in most high-income countries should substantially reduce stillbirth rates in low- and middle-income countries with poorly developed health systems [3,12].

In this supplement to *BMC Pregnancy & Childbirth*, a team of distinguished investigators led by Drs Zulfiqar Bhutta (Aga Khan University), Gary Darmstadt (Johns Hopkins University), and Joy Lawn (Saving Newborn Lives/Save the Children) and also including investigators from Aga Khan University (MY Yakoob, T Soomro, EV Menezes) and Johns Hopkins University (RA Haws), have presented an overview of the stillbirth issue and undertaken a thorough evaluation of the interventions that might potentially reduce their numbers, with a focus on low- and middle-income countries [13-18]. As the authors note, stillbirths are one of the most common adverse pregnancy outcomes, with numbers approximately equivalent to neonatal deaths, post-neonatal infant deaths, childhood deaths (1 to 5 years of age) and adult deaths due to AIDS [13]. Unlike these other mortalities, stillbirth has received little attention, and as an example, is not formally included in any of the global indicators of disease. Interventions to reduce stillbirth are rarely studied, and those interventions evaluated to improve other indicators of maternal and newborn health have rarely included an evaluation of their impact on stillbirth. A formal and detailed review of the literature on potential interventions that might reduce stillbirth is an important first step in choosing the interventions that might play a significant role in addressing this important and understudied problem.

This literature review assumes that the important causes of stillbirth are known and, if only these conditions were targeted with appropriate screening and treatment, substantial reductions in stillbirth would occur. In a general sense, this assumption is correct. Among the major killers of fetuses worldwide are: 1) obstructed labour and the ensuing trauma, asphyxia and infections, 2) infections not related to obstructed labour such as syphilis and malaria, 3) asphyxia associated with maternal and fetal complications including poor placental function, 4) maternal haemorrhage predominantly caused by placental abruption, 5) severe pre-eclampsia and eclampsia, as well as 6) maternal/fetal malnutrition, 7) congenital anomalies and 8) umbilical cord complications [5].

Important differences in the contribution of these various causes are seen between geographic areas, but in general, in every location, these are the major killers. Nevertheless, it is important for each country that is attempting to lower its stillbirth rate to understand the local causes of fetal death so that appropriate screening and treatment strategies can be developed and implemented. In many high-income countries, fetal autopsies and placental histological examination, in addition to the medical history, have been evaluated together to designate a cause of death [19]. Even when all of this information is available, about a third of all stillbirths have no definitive cause. In many low- and middle-income countries, autopsies are almost never available and placental examinations are rarely performed – thus the exact cause of death is rarely known with any degree of certainty. Studies are currently underway to evaluate the effectiveness of verbal autopsy in which structured interviews of the mother, family and birth attendants are used to determine cause of death [8,20-22]. Whether this technique will have sufficient accuracy for determining the correct cause of fetal death compared to autopsy and placental examination is unknown.

Two of the major problems in developing an accurate understanding of stillbirths worldwide are the paucity of vital statistic data for stillbirth from many countries and the lack of consistency in available data over time or between geographic areas. Many reports, for example, present only perinatal mortality data and do not distinguish between stillbirth and neonatal mortality. In the first paper in this series, Lawn et al describe the many different birth weight and gestational age cut-offs that have been used to define a stillbirth. This leads to the inability to make meaningful international comparisons between geographic areas or to know the full extent of the problem worldwide. For this series, the authors have agreed to use the commonly accepted international standards of 28 weeks or 1000 g as the lower gestational age and birth weight cut-offs. We should note that most states in the US use 20 weeks as the lower gestational age cut-off, and that in the US, half of all stillbirths occur between 20 and 28 weeks (or are less than 1000 g) [2]. If these numbers represent the contribution of 20 to 28 week pre-term births to stillbirth rates worldwide, substantially more stillbirths – defined as 20 weeks or more – occur than the 3.2 million annual stillbirths quoted in this series. Adjusting global estimates on this assumption suggests a figure of 6 million stillbirths per year or more worldwide.

We have been particularly interested in the relationship between various infections and stillbirth. Our review suggests that 10 to 25% of stillbirths in high-income countries are caused by infections, while a larger percentage of a much greater number of stillbirths are likely caused by infection in low- and middle-income countries [23]. We have been impressed by the large diversity and number of organisms that are reported to cause stillbirths and especially the wide variety of these types of organisms associated with vector and animal-borne infections. These have never been studied in any systematic way and their numerical contribution to the aetiology of stillbirth is unknown. Nevertheless, the recommendations from this series of papers to focus on syphilis and malaria seem to be directly on target.

Overall, more than 50% of the early stillbirths in all settings, and smaller but important components of the late pre-term and term stillbirths, are associated with histologic chorioamnionitis. Chorioamnionitis – or inflammation of the fetal membranes – is caused by more than 50 different organisms, and among the most common are *Ureaplasma urealyticum*, *Mycoplasma hominis, Escherichia coli*, and Group B streptococcus. To date, there are few strategies that have been consistently shown to reduce chorioamnionitis and the pre-term births and stillbirths associated with this condition. Reducing chorioamnionitis should be the focus of a major research effort as should a vigorous attempt to identify the contribution of all infections to stillbirths using modern molecular techniques, especially in various low- and middle-income country populations.

Finally, it should be clear that stillbirths do not occur in isolation from other adverse perinatal outcomes [24]. High rates of maternal mortality and fistulas as well as neonatal deaths and long-term childhood morbidity all tend to co-occur in the same populations and geographic areas. Interventions that reduce stillbirths are also likely to reduce the other perinatal morbidities and interventions that successfully reduce maternal and early neonatal mortality should reduce stillbirths as well. While not specifically tested as an intervention that reduces stillbirth, the interventions known collectively as emergency obstetric care, which focuses on timely Cesarean section, along with other interventions to reduce maternal deaths, should have an important impact on stillbirths as well [11]. Based on the thorough and comprehensive review done by the authors of this series, we now know many of the evidence-based interventions that work to reduce stillbirths. As importantly, the authors of this series have defined the practices that are likely to have little or no impact on stillbirth rates. Continuing the use of these practices, if nothing else, consumes resources that could be better used to support interventions that work. Even within low-resource settings, large disparities in access to life-saving care, such as Cesarean sections, exist between the richest and poorest women [25]. We now need to make interventions that are of proven value in reducing stillbirths widely available and their use sustainable, especially in these settings. Countries or geographic areas without a developed health care system will almost always need the creation of some basic health care infrastructure to establish a setting where interventions of proven value can be introduced.

The work that led to the articles in this series was funded by the Bill & Melinda Gates Foundation through Saving Newborn Lives because of the desire of both organizations to more clearly define the evidence base for programs that attempt to reduce stillbirths in both high-income and low- and middle-income country settings. While the authors have identified a number of interventions that if widely implemented should substantially reduce the number of stillbirths worldwide, they have also made it clear that there are many areas that would benefit from additional research. One contribution of research is the development and testing of innovative and effective interventions. Another is programmatic research that will teach all of us how to scale up interventions known to be beneficial and to implement stillbirth reduction programs in a sustainable cost-effective way in areas where the burden of stillbirth is high and the resources available are limited.

## Competing interests

The authors declare that they have no competing interests.
